# Sunitinib reduces the infection of SARS-CoV, MERS-CoV and SARS-CoV-2 partially by inhibiting AP2M1 phosphorylation

**DOI:** 10.1038/s41421-020-00217-2

**Published:** 2020-10-13

**Authors:** Pei-Gang Wang, Dong-Jiang Tang, Zhan Hua, Zai Wang, Jing An

**Affiliations:** 1 Department of Microbiology, School of Basic Medical Sciences, Capital Medical University, Beijing, 100069 China; 2 Zhuhai SanMed Biotech Ltd, Zhuhai, 519000 China; 3 Joint Research Center of Liquid Biopsy in Guangdong, Hong Kong and Macau, Zhuhai, 519000 China; 4 Department of General Surgery, China-Japan Friendship Hospital, 100029 Beijing, China; 5 Institute of Clinical Medical Sciences, China-Japan Friendship Hospital, 100029 Beijing, China

**Keywords:** Endocytosis, Membrane fusion

Dear Editor,

In the past 17 years, coronaviruses including SARS-CoV, MERS-CoV and SARS-CoV-2 have crossed the species barrier and resulted in remarkable epidemics in human for three times. Each disease caused by them, especially COVID-19 that is caused by SARS-CoV-2^1^, led to tremendous life threatening and economic loss. There is no effective treatment against them currently, and the development of druggable target is urgently needed. Considering the frequent invasion into human by various coronaviruses, broad spectrum drugs against coronaviruses are particularly important.

HIV backbone-based pseudotyped virus carries a luciferase reporter gene, which is a safe and convenient tool to study the entry of highly virulent pathogens such as SARS-CoV and MERS-CoV. Using this tool, we have previously identified ACE2 as the receptor of SARS-CoV^2^, and analyzed the immunoreactivity of the sera from MERS-CoV-infected animals^3^. In the current study, we used SARS-CoV pseudotyped virus (HIV/SARS-CoV pseudovirus) to screen a siRNA library, and identified AP2M1 as a crucial host factor for SARS-CoV infection. Based on the discovery, we further demonstrated that sunitinib, a kinase inhibitor involving in the regulation of AP2M1, not only inhibited the entry of HIV/SARS-CoV pseudovirus, but also functioned on SARS-CoV-2 and MERS-CoV, thus held great potential as an anti-coronavirus drug.

The siRNA library used for screening is an intracellular membrane traffic siRNA library targeting 144 host molecules, and the primary screening results suggested that AP2M1 may play an important role in SARS-CoV infection. AP2M1 encodes the μ2 subunit of AP2 complex, which is an adapter protein complex for clathrin. AP2M1, clathrin as well as some other factors constitute a clathrin-dependent endocytic pathway by which cells absorb metabolites, hormones, proteins—as well as some viruses—by the inward budding of the plasma membrane. To validate the function of AP2M1 in coronavirus entry, we used two siRNAs to knock down AP2M1 expression (Fig. 1a), and then analyzed the impact on SARS-CoV pseudovirus infection. Neither of the two siRNAs showed cytotoxicity in transfected cells, as revealed by CCK8 assay (Supplementary Fig. S1). In cells transfected with these two siRNAs, the infectivity of HIV/SARS-CoV was significantly reduced to a similar level as HIV/VSV, which was used as a control in the experiment (Fig. 1b). Next, we examined the effect of chlorpromazine (CPZ), the inhibitor of clathrin-mediated endocytosis, on pseudotyped virus infection. CPZ effectively inhibited the infection of HIV/SARS-CoV in a dose-dependent manner, but had no effect on the infection of HIV/AMLV which entered cells in a clathrin-independent way (Fig. 1c), showing that SARS-CoV infection depended on clathrin-mediated endocytosis.

**Fig. 1:**
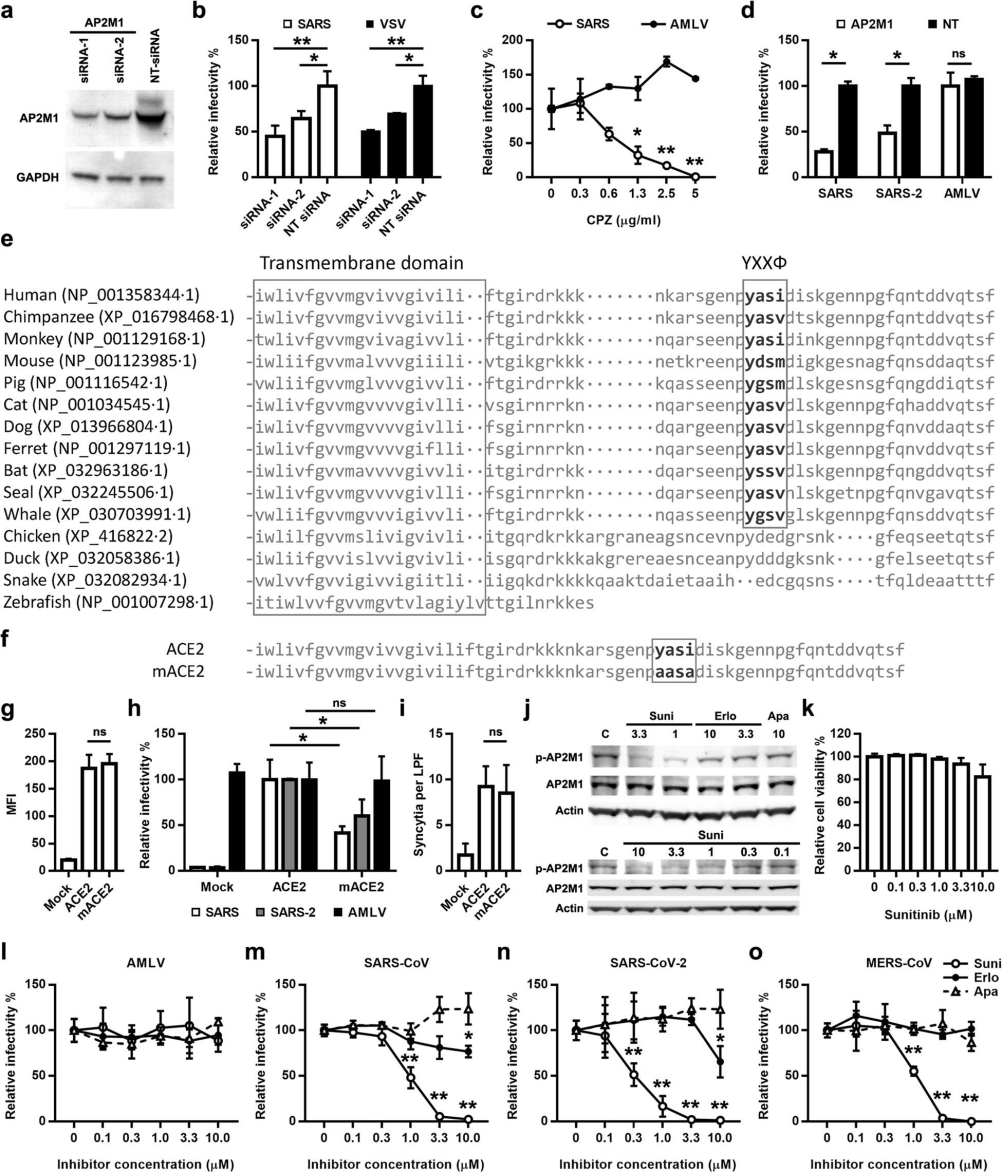
AP2M1 is essential in coronavirus entry and can be targeted by kinase inhibitors. **a** Protein levels of AP2M1 in ACE2-HeLa cells transfected with siRNA-1 and siRNA-2 targeting AP2M1 and NT siRNA examined by western blot. NT non-targeting. **b** Relative infectivity of HIV/SARS-CoV and HIV/VSV on ACE2-HeLa cells transfected with siRNA-1, siRNA-2 and NT siRNA. The infection of pseudovirus was determined by measuring the luciferase activity, and expressed as relative infectivity compared with the control. **c** Relative infectivity of HIV/SARS-CoV and HIV/AMLV on ACE2-HeLa cells treated with different concentrations of CPZ. **d** Relative infectivity of HIV/SARS-CoV, HIV/SARS-CoV-2 and HIV/AMLV on ACE2-HeLa cells transfected with siAP2M1 (siRNA-2) or NT siRNA. **e** Sequence alignment of transmembrane domain and cytoplasmic tail of ACE2 protein in different species. **f** Mutation of YXXΦ motif in mACE2 construct. **g** Surface expression levels of ACE2 on ACE2-HeLa and mACE2-HeLa cells as determined by flow cytometry. **h** Relative infectivity of HIV/SARS-CoV, HIV/SARS-CoV-2 and HIV/AMLV on ACE2-HeLa and mACE2-HeLa cells. **i** Syncytia formation of HeLa cells expressing the S protein of SARS-CoV with ACE2-HeLa or mACE2-HeLa. **j** AP2M1 and phosphorylated AP2M1 levels in ACE2-HeLa cells treated with sunitinib, erlotinib or apatinib. **k** Cytotoxicity of sunitinib on ACE2-HeLa cells. **l**–**o** Relative infectivity of HIV/AMLV (**l**), HIV/SARS-CoV (**m**), HIV/SARS-CoV-2 (**n**), or HIV/MERS-CoV (**o**) on target cells treated with sunitinib, erlotinib or apatinib. Data were derived from three independent experiments, shown as mean ± SD. **P < 0.01; *P < 0.05; ns not significant.

As SARS-CoV and SARS-CoV-2 both utilize ACE2 as the receptor for viral entry^1,2,4^, we asked whether AP2M1 functions for SARS-CoV-2. SARS-CoV-2 pseudovirus system was similarly established and used to test the effect of siAP2M1 in viral entry. The result showed that knock down of AP2M1 inhibited the infectivity of HIV/SARS-CoV and HIV/SARS-CoV-2 but not HIV/AMLV (Fig. 1d), suggesting potential interaction between ACE2 and AP2M1.

There are at least 20 clathrin adaptor proteins, and each adaptor is generally considered to be involved in a different trafficking step. Specifically, AP2 functions in assisting proteins in plasma membrane to be incorporated into endocytic clathrin-coated vesicles. By recognizing YXXΦ (Φ is a hydrophobic residue—L/I/M/V/F) or dileucine motifs, AP2M1 links the cytoplasmic tail of a transmembrane protein to clathrin^5^. To interrogate the hypothesis that AP2M1 may interact with ACE2 and mediated clathrin-mediated endocytosis, we analyzed the sequence of ACE2 and found that a YASI sequence, fulfilling the requirement of the YXXФ element, was presented in the middle of the cytoplastic region of ACE2. Further analysis showed that this motif is conserved among mammalian species including aquatic mammals and bat, which is believed to be the natural host of SARS-CoV (Fig. 1e).

To analyze whether the YASI motif was essential for SARS-CoV entry, we replaced the coding sequence of YASI with AASA in the ACE2 expression vector (Fig. 1f), and established HeLa cells stably expressing the mutant ACE2 (mACE2-HeLa) by retroviral vectors. Replacing YASI with AASA did not affect the surface expression level of ACE2 on HeLa cells (Fig. 1g). However, compared with HeLa cells expressing wild-type ACE2 (ACE2-HeLa), the sensitivity of mACE2-HeLa cells to HIV/SARS-CoV or HIV/SARS-CoV-2 infection was significantly reduced, but the susceptibility to HIV/AMLV did not change (Fig. 1h), suggesting that the potential interaction between AP2M1 and YASI motif in ACE2 plays an important role during the entry of SARS-CoV and SARS-CoV-2 into host cells.

We also observed the effect of mACE2 on cell-cell fusion. ACE2 or mACE2-expressing cells were metabolically labeled with CMFDA and co-cultured with SARS-CoV spike protein (S) expressing cells, which were separately metabolically labeled with Octadecyl Rhodamine B Chloride (R18). CMFDA and R18 are green and red fluorescence dyes respectively. The cell-cell fusion mediated by spike-ACE2 interaction was thus quantified by counting the number of syncytia which were co-stained with two fluorescence dyes. Compared with HeLa-ACE2 cells, mACE2-HeLa cells showed no difference in the ability to form syncytia with HeLa cells expressing the spike protein of SARS-CoV (Fig. 1i), implying that the mutation of AP2M1 recognition element in ACE2 does not affect its ability to interact with spike protein and cause viral-cell membrane fusion at cell surface.

AP2M1 has to be phosphorylated at Thr156 before it recognizes and binds to the YXXΦ motif and initiates the following internalization of membrane cargos^5^. The Thr156 phosphorylation of AP2M1 favors a new, cargo-bound conformation of AP2 and simultaneously creates a binding platform for the endocytic proteins^6^. ARK family kinase AAK1, and occasionally NAK family kinase GAK, are responsible for the phosphorylation of AP2M1, making them potential molecule targets for anti-viral therapy. As AAK1 and GAK can be targeted by sunitinib and erlotinib respectively^7^—both are multi-target kinase inhibitors as anti-tumor drugs in clinical use—we examined the effects of these drugs on pseudoviral entry. Apatinib, which inhibits VEGFR2 with high specificity, was used as a control. As shown in western blot (Fig. 1j), sunitinib significantly inhibited the phosphorylation of AP2M1 at 1 μM, with no apparent effect on cellular viability measured by CCK8 assay (Fig. 1k). Western blot with treatment of sunitinib ranging from 0.1 to 10 μM further showed a dose-dependency of sunitinib’s inhibitory effects on AP2M1 phosphorylation (Fig. 1j, lower panels). In contrast, erlotinib only slightly inhibited AP2M1 phosphorylation at 10 μM and apatinib did not inhibit AP2M1 phosphorylation (Fig. 1j).

In line with the inhibition of AP2M1 phosphorylation, sunitinib showed the best inhibitory effect on pseudoviral entry except the AMLV control (Fig. 1l). For SARS-CoV pseudoviruses, sunitinib significantly inhibited the infection at 1 μM (Fig. 1m); for SARS-CoV-2, sunitinib even functions at 0.33 μM (Fig. 1n) that can be achieved and maintained for 4-6 hours in patients who take 75–100 mg sunitinib daily^8^. Whereas erlotinib only inhibited SARS-CoV and SARS-CoV-2 at 10 μM (Fig. 1m, n). As a negative control, Apatinib had no inhibitory effect at any concentration tested (Fig. 1m, n).

Intriguingly, when sunitinib was tried on MERS-CoV pseudovirus, it similarly inhibited the infection (Fig. 1o), suggesting sunitinib is likely to be an inhibitor for dangerous coronaviruses infection with broad spectrum. MERS-CoV utilizes DPP4 as its receptor^9^. Unlike ACE2, DPP4 is a type II transmembrane protein and has a very short cytoplasmic region (MKTPWK) at its N terminus. As no recognizable motif for AP2M1 is present in the cytoplasmic region of DPP4, sunitinib inhibition on MERS-CoV might be distinct from that of SARS-CoV and SARS-CoV-2, and lots of work needs to be done before the mechanism is revealed.

Very recently, baricitinib, which is a JAK1 and JAK2 inhibitor and has been used for therapy of rheumatoid arthritis, has been suggested as a promising antiviral drug for SARS-CoV-2^10^. Since baricitinib has been predicted to be an inhibitor for AAK1^11^, it was also tried on three types of pseudovirus. Unexpectedly, although baricitinib has been shown to own high affinity to AAK1, it did not display obvious inhibition on the phosphorylation of AP2M1 unless at very high dose (10 μM) (Supplementary Fig. S2a), and consequently displayed no inhibitory effect on infection of each type of pseudovirus (Supplementary Fig. S2b). Nevertheless, this result only implied that AP2M1 was not associated with baricitinib. As baricitinib can inhibit JAK1 and JAK2 signaling pathway, it may be helpful for COVID-19 therapy by inhibiting inflammation^11^.

Taken together, we identified AP2M1 as a crucial host factor for coronaviral entry and can be targeted by kinase inhibitors like sunitinib. AP2M1 interacts with YASI sequence in the cytoplastic tail of ACE2, and mediates clathrin-dependent entry for SARS-CoV. While SARS-CoV-2 also uses ACE2 as receptor^1^, the function of AP2M1 in SARS-CoV-2 entry may be similar to that in SARS-CoV entry. Based on the discovery of AP2M1, sunitinib was identified as a potential coronaviruses inhibitor with broad spectrum. Besides coronavirus, sunitinib also inhibits viral entry including HCV^12^, rabies virus^13^, dengue virus, and Ebola virus^14^, suggesting that its application in anti-viral research should be paid more attention.

In spite of these advances, we have to kept two issues in mind. First, besides clathrin-dependent pathway, SARS-CoV, MERS-CoV and SARS-CoV-2 also utilizes TMPRSS2 to cleave its S protein and initiates a direct viral-cell membrane fusion to enter cells^15–17^, suggesting the importance of combination of sunitinib and TMPRSS2 inhibitor^17^. Second, the clathrin-dependent endocytosis is crucial for many cellular singling pathways, and its disruption in general might lead to severe cytotoxicity and side effects. The therapy effects and side effects, therefore, should be carefully balanced before the application of these inhibitors.

## Supplementary information

Revised Supplementary File

## Supplementary information

**Supplementary Information** accompanies the paper at (10.1038/s41421-020-00217-2).
