# Supplementary material for: Sunitinib reduces the infection of SARS-CoV, MERS-CoV and SARS-CoV-2 partially by inhibiting AP2M1 phosphorylation
Source: Cell Discov. 2020 Oct 13;6:71. doi: 10.1038/s41421-020-00217-2 (PMC7550610; doi:10.1038/s41421-020-00217-2)
Supplement: Supplementary file 1 — Revised Supplementary File [file 41421_2020_217_MOESM1_ESM.docx]

**Supplementary information**

**Supplementary Figures**


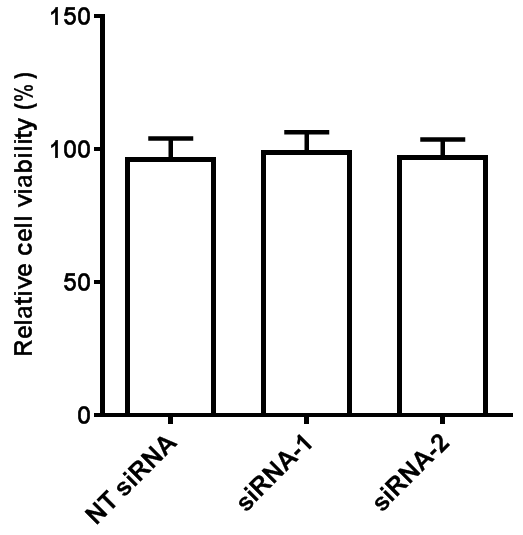


Supplementary Fig. 1: Cytotoxicity of AP2M1 siRNAs on ACE2-HeLa cells. ACE2-HeLa cells were transfected with two siRNAs targeting AP2M1 or non-targeting siRNA as control. Cell viability was measured by a CCK-8 assay and expressed as mean ± SD.


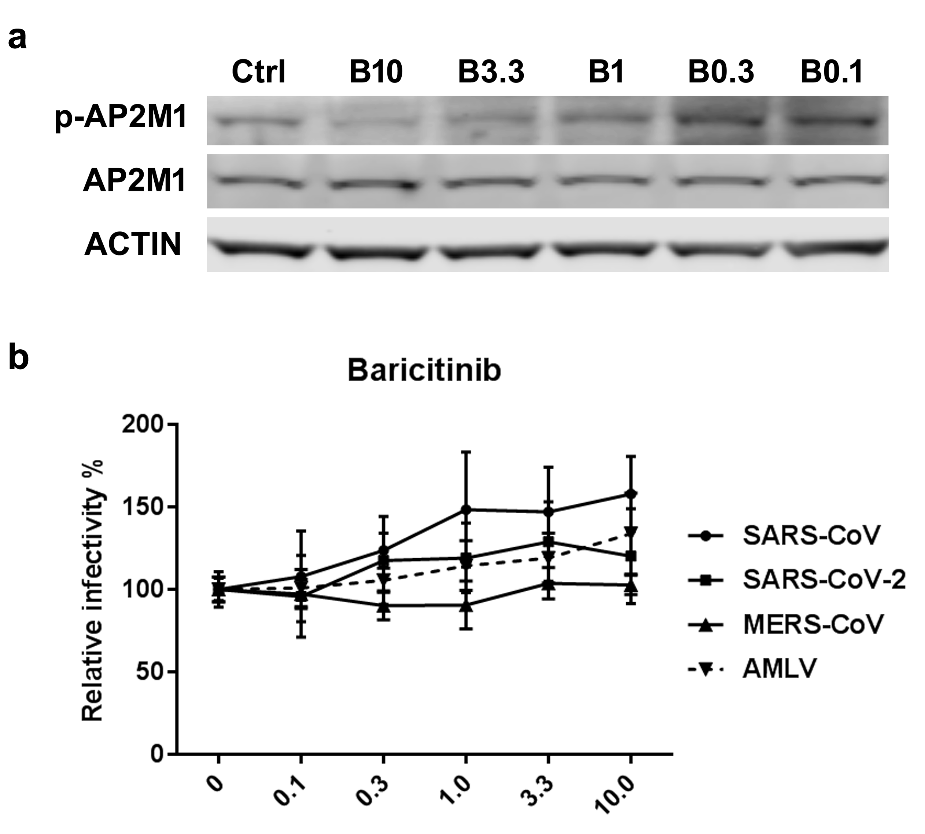


Supplementary Fig. 2: Effects of baricitinib on AP2M1 phosphorylation and pseudoviruses infection. a) AP2M1 and phosphorylated AP2M1 levels in ACE2-HeLa cells treated with baricitinib at various concentrations. b) Relative infectivity of HIV/AMLV, HIV/SARS-CoV, HIV/SARS-CoV-2, or HIV/MERS-CoV on target cells treated with baricitinib at various concentrations. Data were derived from three independent experiments, shown as mean ± SD.

**Materials and Methods**

**Constructions**

Wild type human ACE2, as well as SARS-CoV Spike gene (S), were cloned as previously described ^1^. The YASI motif at the C terminal of ACE2 was changed into AASA by over-lap PCR using wild type human ACE2 as template. Primers used in the PCR are forward 1: 5’-CCCTTATTTACCTGGCTGAAAGAC-3’, reverse 1: 5’-CACCCTAACTGACACACATTCCAC-3’, forward 2: 5’-GCTGCCTCCGCCGATATTAGCAAAGGAGAAAATAATC-3’ and reverse 2: 5’-TATCGGCGGAGGCAGCAGGATTTTCTCCACTTCTTGC-3’. Wild type, mutant ACE2 and S genes were subcloned into pBabepuro, a retroviral vector and separately transfected into HeLa cells as previously described ^1^. Cells that expressing target genes were selected by culture in medium containing 10 μg/ml puromycin for 1 week.

**Cell lines**

293T, Huh7.5, HeLa and its derivative cells were maintained in DMEM supplemented with 10% fetal bovine serum (FBS) and 1% penicillin/streptomycin at 37 °C with 5% CO2.

**Pseudotyped virus infection assay**

The codon-optimized spike genes of SARS-CoV and MERS-CoV were described as previous reports ^1,2^. The spike gene of SARS-CoV-2 (Wuhan-Hu-1 strain) was codon-optimized and cloned into pcDNA3.1(+) with C-terminal 19-aa deletion ^3^ to generate pcDNA-SARS2-S. To produce HIV/SARS-CoV, HIV/MERS-CoV or HIV/SARS-CoV-2 pseudoparticles, 10 μg pNL Luc E- R- and 10 μg pcDNA-S were co-transfected into 4 x 10^6^ 293T cells. The medium was replaced with fresh medium, and the supernatants of transfected cells were harvested 48 hours post transfection and passed through a 0.45 μM filter (Millipore). The pseudotyped virus was normalized by p24 ELISA using a Vironostika HIV-1 Antigen MicroELISA Kit (Biomerieux bv, Boxtel, The Netherlands). The supernatant containing 5 ng pseudotyped virus (p24) and 8 μg/ml polybrene was used to infect cells in 24-well plates (4 x 10^4^ cells/well). The cells were lysed at 48 hours post infection. 10 μl lysate was tested for luciferase activity by the addition of 50 μl luciferase substrate (Promega) and measured in a Spark^®^ multimode microplate reader (Tecan, Switzerland).

In RNA interference or drug inhibition experiments, the infectivity of pseudovirus was determined by measuring luciferase activity, and expressed relative to that of the control.

**RNA interference**

siRNAs (200 nM) were transfected into cells using Lipofectamine 2000 (Thermo Fisher Scientific) 48 hours before infection. NT siRNA: UUCUCCGAACGUGUCACGU; AP2M1 siRNA: siRNA1: UAUAUGAGCUGCUGGAUGA; siRNA2: CGUGAUGGCUGCCUACUUU.

**Western blotting**

Cells were lysed in SDS loading buffer and the lysates were subjected to 4-20% PAGE gel (Yeasen), transferred onto PVDF Immobilon^®^-Psq transfer membrane (Millipore). Blots were blocked with Odyssey^®^ Blocking Buffer (TBS) (LI-COR Biosciences) and blotted with rabbit anti-AP1M1 (Abcam), rabbit anti–phosphorylated AP2M1 (Abcam) and mouse anti–β-actin (Thermo Fisher Scientific) antibodies. Secondary antibodies used were IRDye^®^ 800CW Goat anti-Mouse IgG and IRDye^®^ 680RD Goat anti-Rabbit IgG (LI-COR Biosciences). The blots were imaged on the Odyssey^®^ CLx imaging system using 700 nm and 800 nm channels and analyzed using ImageStudio software (LI-COR Biosciences).

**Flow cytometry**

HeLa cells expressing wild type or mutant ACE2 were detached by incubation in 10 mM EDTA at 37℃ for 10 min. After washing, the cells were incubated with an anti-ACE2 antibody (Abcam) for 1 hour at 4℃. Isotype IgGs (Abcam) were used as controls. Cells were then washed and incubated for 30 minutes with a fluorescein isothiocyanate (FITC)-conjugated secondary antibody (1:200; Abcam). Cells were analyzed using a Facscalibur flow cytometer (BD Biosciences, San Jose, CA, USA).

**Cell fusion assay**

ACE2 or mACE2-expressing cells and SARS-CoV spike expressing cells were metabolically labelled with CellTracker™ Green CMFDA Dye (Thermofisher) or Octadecyl Rhodamine B Chloride (Thermofisher) as described in the main text. These cells were then co-cultured (1:1) at 37 °C for 6 hours. The cell-cell fusion mediated by spike-ACE2 interaction was quantified by counting the number of syncytia under the microscope.

**Pharmacological inhibition assay**

Cells were treated with 10, 3.3, 1, 0.33, 0.1 μM sunitinib malate (Aladdin, Shanghai, China), erlotinib HCL (Selleck, Houston, TX, USA), baricitinib (Selleck, Houston, TX, USA) or apatinib mesylate (HengRui Medicine Co. LTD, Jiangsu, China) for 1 hour at 37 ℃ before and for the duration of infection.

**Cell viability assays**

Cell viability was measured by a Cell Counting Kit-8 (CCK-8) (WST-8, Dojindo, Kumamoto, Japan). Cells were cultured in 96-well plates (7.5 × 10^3^ cells/well) 24 hours before treated with different drugs or transfected with siRNAs as indicated. 48 hours after treatment, 10 μl WST-8 reagent solution was added to each well, and the plate was incubated for 2 hours at 37°C. Absorbance was measured at 450 nm using a microplate reader (Thermo Electron Corporation, Inc., Waltham, MA, USA).

**Statistical analysis**

Student's t‑test was used to compare the statistical difference between two groups. For data containing multiple groups, statistical analyses were performed using one‑way analysis of variance followed by the Tukey's post hoc test using GraphPad Prism software (version 6.01; GraphPad Software, Inc.).

**References**

1 Bekerman, E. *et al.* Anticancer kinase inhibitors impair intracellular viral trafficking and exert broad-spectrum antiviral effects. *J Clin Invest* **127**, 1338-1352, doi:10.1172/JCI89857 (2017).

2 Perera, R. A. *et al.* Seroepidemiology for MERS coronavirus using microneutralisation and pseudoparticle virus neutralisation assays reveal a high prevalence of antibody in dromedary camels in Egypt, June 2013. *Euro Surveill* **18**, pii=20574, doi:10.2807/1560-7917.es2013.18.36.20574 (2013).

3 Ou, X. *et al.* Characterization of spike glycoprotein of SARS-CoV-2 on virus entry and its immune cross-reactivity with SARS-CoV. *Nat Commun* **11**, 1620, doi:10.1038/s41467-020-15562-9 (2020).
